# Does Cannabis Composition Matter? Differential Effects of Delta-9-tetrahydrocannabinol and Cannabidiol on Human Cognition

**DOI:** 10.1007/s40429-017-0142-2

**Published:** 2017-04-29

**Authors:** Marco Colizzi, Sagnik Bhattacharyya

**Affiliations:** Department of Psychosis Studies, Institute of Psychiatry, Psychology and Neuroscience, King’s College London, London, SE5 8AF UK

**Keywords:** Cannabis, Delta-9-tetrahydrocannabinol, Cannabidiol, Cognitive processing, Brain function

## Abstract

**Purpose of Review:**

The lack of clarity about the effect of cannabis use on cognition may be attributable to the considerable heterogeneity among studies in terms of cannabis composition. This article selectively reviews studies examining the distinctive effects of cannabinoids on human cognition, particularly those of delta-9-tetrahydrocannabinol (Δ9-THC) and cannabidiol (CBD).

**Recent Findings:**

Research indicates that ∆9-THC administration acutely impairs cognition, particularly memory and emotional processing. Limited evidence suggests that CBD administration might improve cognition in cannabis users but not in individuals with neuropsychiatric disorders. Moreover, studies indicate that some acute Δ9-THC-induced cognitive impairments may be prevented if Δ9-THC is administered in combination or following CBD treatment. Δ9-THC and CBD have also shown opposite effects on cognition-related brain activation, possibly reflecting their antagonistic behavioral effects.

**Summary:**

Research suggests greater cognitive impairments in individuals when exposed to high ∆9-THC or low CBD cannabis. It is unclear whether at specific concentrations CBD might outweigh any harmful effects of Δ9-THC on cognition.

## Introduction

Interest in the effects of cannabis on human cognition stems from evidence regarding its role as the most widely used illicit drug [1] as well as an important risk factor for the development of psychosis [2, 3] and its relapse [4–8], the latter being also consistent with evidence that delta-9-tetrahydrocannabinol (∆9-THC), the main psychoactive ingredient of the *Cannabis sativa* plant, can induce transient psychotic symptoms upon acute administration in healthy volunteers [9–11]. This body of research is also consistent with independent evidence of endocannabinoid system abnormalities in psychosis [12], a disorder characterized by abnormalities in different cognitive processes [13].

However, evidence regarding the association between cannabis use and impairments in cognitive processing is less clear. While one may intuitively expect cannabis to have a deleterious effect on cognitive performance, studies in healthy volunteers have reported some discrepancies. For example, some studies suggest that healthy cannabis users show poorer cognitive performance [14–17]. Others report no difference in cognitive processing as a function of cannabis use [18]. Even more conflicting results are present in studies in patients with schizophrenia. While some studies suggest poorer cognitive functions in patients with a history of cannabis exposure [19], others indicate better performance [15, 20] or no change [17, 21] in cannabis-using patients.

Variation in the results of studies investigating the effect of cannabis use on cognitive functioning, as outlined above, might have several explanations. These discrepancies may be because of genetic variation having an impact on cognitive phenotypes [22–29]. Moreover, it may reflect selective effect on certain aspects of cognition as suggested by available evidence, with a robust but modest deleterious impact on global memory function, a more pronounced detrimental effect on specific memory sub-domains such as prospective and retrospective verbal and visual memory, and limited effect on other cognitive domains [17, 30]. It may also reflect variation between different studies in the duration of cannabis exposure and/or of abstinence at the time of cognitive testing of study participants, as available evidence points towards a dose-dependent effect with cognitive impairment being more marked following persistent cannabis use and incomplete restoration of function following cessation of use [31]. Evidence from imaging studies also suggests that both chronic [32, 33] and acute cannabis exposure [34] might disrupt activity in brain networks involved in cognitive processing without necessarily affecting behavioral performance, suggesting either a deployment of greater neural effort or a change in strategy to maintain adequate task performance [35], thereby accounting for different effects in different individuals.

Independent of these potential explanations, the lack of a clear association between cannabis use and impairments in cognition may also to a large extent be attributable to the considerable heterogeneity in recreational cannabis that participants in these studies may have been exposed to, as well as the differing effects on cognition of the various chemicals found in the extract of the cannabis plant. The extract of *Cannabis sativa* has over 60 different cannabinoids [36], with ∆9-THC and cannabidiol (CBD) being the most prominent among them. However, while ∆9-THC is thought to be responsible for most of its psychotropic effects [37], CBD is under investigation for its potential antipsychotic effects, in opposition to the pro-psychotic effects of ∆9-THC. Research also suggests that CBD can counteract the negative effects of ∆9-THC, as investigated in both humans and animal models at a behavioral and neurochemical level [38–41]. This is of crucial importance considering that case-control studies suggest that the risk of development and relapse of psychosis in cannabis users depends on both frequency of use and cannabis potency [5, 42], with the risk being the highest in individuals exposed on a daily basis to cannabis with a high ∆9-THC concentration, and unchanged among users of cannabis with a lower ∆9-THC concentration and a more balanced ∆9-THC:CBD ratio. In line with evidence from human studies, research investigating the effect of different cannabinoids in animal models has consistently reported behavioral abnormalities following Δ9-THC exposure. Δ9-THC exposure during adolescence has been associated with long-term behavioral alterations in adult rats, such as recognition memory deficits, social withdrawal, and altered emotional reactivity [43]. Other evidence suggests enduring cognitive impairment in the offspring of rats exposed to Δ9-THC during the perinatal period [44]. Interestingly, altered behavior and cognition in animal models may be directly related to the Δ9-THC-induced dysfunction of the glutamatergic and noradrenergic systems via cannabinoid receptor 1 activation, and this altered neurotransmission can be prevented or reversed if CBD is administered before or after Δ9-THC exposure, respectively [40, 43, 44].

Together, these findings underscore how the effects of different cannabinoids, which are often present in varying concentration in the cannabis available for use in the street, may confound the results of human studies investigating cognitive alterations associated with recreational cannabis use. In this article, we carry out a narrative review of studies examining the acute effects of cannabis on human cognition and related brain function, with emphasis on the distinctive effects of the different cannabinoids that have been examined to date, particularly Δ9-THC and CBD, in order to disentangle their contribution to specific cognitive processes.

## Methods

In order to disentangle the effect of different cannabinoids on cognitive domains relevant to neuropsychiatric disorders, this literature review aimed to mainly focus on human studies that examined the impact of Δ9-THC in contrast with CBD and other cannabinoids on cognitive functioning using cannabis/cannabinoid challenge paradigms. Functional magnetic resonance imaging (fMRI) studies examining the neural correlates of the effects of Δ9-THC and CBD on human cognition and the role of other cannabinoids in modulating cognitive processes during a cannabis/Δ9-THC challenge are also discussed.

### Search Strategy

A literature search was performed using electronic databases (MEDLINE, Web of Science, and Scopus) for original English-language research articles published over the last 25 years (1990–2016). Keywords included delta-9-tetrahydrocannabinol, cannabidiol, cognition, cognitive dysfunction/impairment, and memory/learning. Reference lists of eligible studies were also screened to identify additional studies.

### Eligibility Criteria

Studies were eligible for inclusion in this review if they had assessed the effect of Δ9-THC *and* CBD or another cannabinoid on cognition during acute challenge investigations. Studies were excluded if they (i) did not assess the effects of Δ9-THC, CBD, or other cannabinoids on cognition in experimental studies; (ii) did not investigate the role of different cannabinoids on cognition; (iii) primarily assessed psychological or psychiatric parameters rather than cognition.

## Results

### Evidence at a Glance

A number of studies have assessed the effect of Δ9-THC alone on cognition [41, 45–49]. However, only a limited body of research has specifically compared the effect of different cannabinoids on human cognition, which has also been reviewed before focusing primarily on neuroimaging studies [41, 49]. These studies have used different experimental designs and studied heterogeneous populations. In particular, eight human studies were identified on the behavioral effects of Δ9-THC and CBD, which investigated (i) cognitive function in cannabis users using products with a high or low Δ9-THC:CBD ratio, in both the intoxicated and unintoxicated state [50, 51]; (ii) cognitive function in healthy subjects following administration of pure Δ9-THC and CBD [52••, 53••]; (iii) cognitive function in healthy subjects following administration of standardized cannabis extracts with defined Δ9-THC:CBD ratios [54, 55]; (iv) cognitive function in human subjects prescribed with medical marijuana (MMJ) containing a low/balanced Δ9-THC:CBD ratio [56, 57].

Similarly, a review carried out in 2014 identified 24 studies which used fMRI paradigms to explore the acute effects of Δ9-THC or CBD on human brain function [49]. Most of these studies have investigated how these cannabinoids modulate brain activity in the resting state or during cognitive processing. However, a limited number of studies have specifically compared the effects of pure Δ9-THC and CBD on the neural substrates of human cognition [58••, 59, 60, 61••].

Finally, only one study has investigated the effect of another cannabinoid, delta-9-tetrahydrocannabivarin (Δ9-THCV), by comparing its effects on cognition with that of pure Δ9-THC in healthy subjects [62] (Tables 1 and 2).

**Table 1:** Studies included in the review

Study	Type of study	Study sample	Cannabinoids investigated	Cognitive domain investigated
Wade et al. 2003 [57]	Randomized, double-blind, cross-over, behavioral study	Patients with neurological disorders	Δ9-THC, Δ9-THC + CBD, CBD	Attention
Roser et al. 2008 [54]	Double-blind, cross-over, EEG study	Healthy participants	Δ9-THC, Δ9-THC + CBD	Attention, processing speed
Morgan et al. 2010 [51]	Naturalistic, behavioral study	Cannabis users	Δ9-THC + CBD	Verbal memory, episodic memory, executive function
Bhattacharyya et al. 2010 [58••]	Randomized, double-blind, cross-over fMRI study	Healthy participants	Δ9-THC, CBD	Verbal memory, emotional processing, executive function, visual and auditory processing
Schoedel et al. 2011 [55]	Randomized, double-blind, cross-over, behavioral study	Recreational cannabis users	Δ9-THC, Δ9-THC + CBD	Attention, processing speed, working memory
Winton-Brown et al. 2011 [59]	Pseudo-randomized, double-blind, cross-over, fMRI study	Healthy participants	Δ9-THC, CBD	Visual and auditory processing
Bhattacharyya et al. 2012 [60]	Randomized, double-blind, cross-over, fMRI study	Healthy participants	Δ9-THC, CBD	Attentional salience processing
Morgan et al. 2012 [50]	Naturalistic, behavioral study	Cannabis users	Δ9-THC + CBD	Verbal memory, episodic memory, recognition memory
Englund et al. 2013 [52••]	Randomized, double-blind, behavioral study	Healthy participants	Δ9-THC, Δ9-THC + CBD	Verbal memory, working memory, executive function
Bhattacharyya et al. 2015 [61••]	Randomized, double-blind, cross-over, fMRI study	Healthy participants	Δ9-THC, CBD	Attentional salience processing
Hindocha et al. 2015 [53••]	Randomized, double-blind, cross-over, behavioral study	Cannabis users	Δ9-THC, Δ9-THC + CBD, CBD	Emotional processing
Gruber et al. 2016 [56]	Longitudinal, behavioral study	Patients certified for medical cannabis	Δ9-THC + CBD	Executive function, processing speed
Englund et al. 2016 [62]	Double-blind, cross-over, behavioral study	Healthy participants	Δ9-THC, Δ9-THC + Δ9-THCV	Verbal memory, working memory

*EEG* electroencephalography, *fMRI* functional magnetic resonance imaging, *Δ9-THC* delta-9-tetrahydrocannabinol, *CBD* cannabidiol, *Δ9-THCV* delta-9-tetrahydrocannabivarin, *+* investigated in combination

**Table 2:** Summary of clinical studies comparing the effects of different cannabinoids on human cognition

Study	Aim of study	Population	*n*; age (years)	Cannabinoid concentration	Exposure	Placebo controlled	Behavioral results	Neuroimaging results
Wade et al. 2003 [57]	Effects of Δ9-THC, CBD, and their combination on attention	Patients with multiple sclerosis (*n* = 18), spinal cord injury (*n* = 4), brachial plexus damage (*n* = 1), and amputation (*n* = 1)	24; 48	1. 2.5 mg Δ9-THC 2. 2.5 mg Δ9-THC +2.5 mg CBD 3. 2.5 mg CBD	Acute challenge with titrated sl. Δ9-THC, CBD, or Δ9-THC + CBD	✓	1. *↓ attention* 2. NS 3. NS	NA
Roser et al. 2008 [54]	Effects of Δ9-THC and Δ9-THC + CBD on attention and processing speed	Healthy subjects	20 (10 M, 10 F); 28.2 ± 3.1	1. 10 mg Δ9-THC 2. 10 mg Δ9-THC +5.4 mg CBD	Acute challenge with 4 po. Δ9-THC or Δ9-THC + CBD cps. over 3 weeks	✓	1. NS 2. NS 1. vs. 2. NS	1. *↓ P300 wave amplitudes* 2. *↓ P300 wave amplitudes*
Morgan et al. 2010 [51]	Effects of cannabis CBD content on verbal memory, episodic memory, and executive function	Cannabis-using subjects with different patterns of cannabis use (low CBD, *n* = 22, cannabis use = 17.1 ± 11.2 days per month; high CBD, *n* = 22, cannabis use = 13.3 ± 11.9 days per month)	44; 21.4 ± 2.0 and 21.55 ± 1.8	<0.14% (low) CBD cannabis vs. >0.75% (high) CBD cannabis (in front of NS different Δ9-THC contents)	a. Unintoxicated state b. Chosen strain smk. during study	✕	a. NS b. *high CBD > low CBD in verbal memory*, NS other domains	NA
Bhattacharyya et al. 2010 [58••]	Effects of Δ9-THC and CBD on verbal memory, emotional processing, executive function, and visual and auditory processing	Healthy subjects with lifetime cannabis use <15 times	15 (15 M); 26.7 ± 5.7	1. 10 mg Δ9-THC 2. 600 mg CBD	Acute challenge with po. singles doses of Δ9-THC or CBD	✓	1. NS 2. NS	*1. vs. 2*. *Opposite effects of Δ9-THC and CBD on brain activity related to verbal memory, emotional processing, executive function, and visual and auditory processing*
Schoedel et al. 2011 [55]	Effects of increasing dosages of Δ9-THC and Δ9-THC + CBD on attention, processing speed, and working memory	Frequent recreational cannabis-using subjects	23 (19 M, 4 F); 19–45	1. 20 or 40 mg Δ9-THC 2. 10.8 mg Δ9-THC + 10 mg CBD, 21.6 mg Δ9-THC + 20 mg CBD, 43.2 Δ9-THC + 40 mg CBD	Acute challenge with om. Δ9-THC or Δ9-THC + CBD at different concentrations	✓	1*. ↓ working memory*, NS other domains 2. NS	NA
Winton-Brown et al. 2011 [59]	Effects of Δ9-THC and CBD on visual and auditory processing	Healthy subjects with lifetime cannabis use <15 times	14 (14 M); 26.7 ± 5.7	1. 10 mg Δ9-THC 2. 600 mg CBD	Acute challenge with po. single doses of Δ9-THC or CBD	✓	NA	*1. vs. 2. Opposite effects of Δ9-THC and CBD on brain activity related to visual and auditory processing*
Bhattacharyya et al. 2012 [60]	Effects of Δ9-THC and CBD on attentional salience processing	Healthy subjects with lifetime cannabis use <15 times	15 (15 M); 26.7 ± 5.7	1. 10 mg Δ9-THC 2. 600 mg CBD	Acute challenge with po. single doses of Δ9-THC or CBD	✓	1. NS, however *Δ9-THC-induced striatal deactivation correlated with response latency* 2. NS	*1. vs. 2. Opposite effects of Δ9-THC and CBD on brain activity related to attentional salience*
Morgan et al. 2012 [50]	Effects of cannabis Δ9-THC and CBD contents on verbal memory, episodic memory, and recognition memory	Cannabis-using subjects with different patterns of cannabis use (recreational use, *n* = 54, cannabis use ≤25 days per month; daily use, *n* = 66, cannabis use ≥25 days per month)	120 (89 M, 31 F); 16–23	1. Low CBD vs. high CBD cannabis 2. Low Δ9-THC cannabis vs. high Δ9-THC cannabis (Δ9-THC and CBD contents tested in hair samples)	Unintoxicated state	✕	1*. high CBD > low CBD in recognition memory*, independently of frequency of use 2. *daily high Δ9-THC < low Δ9-THC in verbal and episodic memory*	NA
Englund et al. 2013 [52••]	Effects of CBD pretreatment prior to Δ9-THC challenge on verbal memory, working memory, and executive function	Healthy subjects (lifetime cannabis use PLB group: 118 ± 218 episodes; lifetime cannabis use CBD group: 137 ± 234 episodes)	48 (27 M, 21 F); 21–50	1. 1.5 mg Δ9-THC 2. 600 mg CBD pretreatment prior to 1.5 mg Δ9-THC	Acute challenge with single doses of iv. Δ9-THC or iv. Δ9-THC + po. CBD	✓	1. *↓ working memory* 2. *Δ9-THC + CBD > Δ9-THC in verbal memory and some working memory components* NS other domains	NA
Bhattacharyya et al. 2015 [61••]	Effects of Δ9-THC and CBD on attentional salience processing	Healthy subjects with lifetime cannabis use <15 times	15 (15 M); 26.7 ± 5.7	1. 10 mg Δ9-THC 2. 600 mg CBD	Acute challenge with po. single doses of Δ9-THC or CBD	✓	1. NS, however *Δ9-THC-induced reduction of fronto-striatal connectivity correlated with response latency* 2. NS	*1. vs. 2. Opposite effects of Δ9-THC and CBD on brain connectivity related to attentional salience*
Hindocha et al. 2015 [53••]	Effects of Δ9-THC, CBD and their combination on emotional processing	Cannabis-using subjects with different patterns of cannabis use (light use, *n* = 24, cannabis use ≤25 days per month; heavy use, *n* = 24, cannabis use ≥25 days per month)	48 (34 M, 14 F); 21 < mean < 23	1. 8 mg Δ9-THC 2. 8 mg Δ9-THC +16 mg CBD 3. 16 mg CBD	Acute challenge with inh. Δ9-THC, CBD, or Δ9-THC + CBD	✓	1*. ↓ emotional processing,* independently of frequency of use and schizotypy traits 2*. Δ9-THC + CBD > Δ9-THC in emotional processing* 3. *↑ emotional processing*, independently of frequency of use and schizotypy traits	NA
Gruber et al. 2016 [56]	Effects of MMJ on executive function and processing speed	MMJ certified patients with anxiety (*n* = 5), depression (*n* = 3), chronic pain (*n* = 7), sleep (*n* = 5), and other conditions (*n* = 6); MMJ use: 9.3 ± 8.8 episodes per week	11 (6 M; 5 F); 48.9 ± 15.1	Δ9-THC + CBD at unspecified dosages	3-month treatment	✕	*↑ executive function and processing speed*	NA
Englund et al. 2016 [62]	Effects of Δ9-THCV pretreatment prior to Δ9-THC challenge on verbal memory and working memory	Healthy subjects with lifetime cannabis use (<25 times)	10 (10 M); 23.8	1. 1 mg Δ9-THC 2. 50 mg Δ9-THCV pretreatment prior to 1.5 mg Δ9-THC	Acute challenge with a single dose of iv. Δ9-THC or a single dose of iv. Δ9-THC + 5 days of po. Δ9-THCV	✓	1. *↓ verbal memory*, NS on other domains 2. *Δ9-THC + Δ9-THCV > Δ9-THC in some verbal memory components but < in others*	NA

*Δ9-THC* delta-9-tetrahydrocannabinol, *CBD* cannabidiol, *Δ9-THCV* delta-9-tetrahydrocannabivarin, *PLB* placebo; *+* investigated in combination, *M* male, *F* female, *sl.* sublingual, *po.* per os, *smk*. smoking, *om.* oromucosal, *iv.* intravenous, *inh.* inhalation, *↓* lower/poorer, *↑* higher/better, *<* lower/poorer, *>* higher/better, *NS* not significant, *NA* not applicable/not assessed

In contrast to studies on the effects of cannabis or Δ9-THC on cognition, there has been very limited examination on the effect of CBD alone on human cognition, with only two studies assessing the effect of CBD on cognitive function in healthy subjects [58••] and in schizophrenia patients [63], respectively. One of those exposed healthy participants to Δ9-THC, CBD, or placebo on separate occasions and is therefore included in this review [58••]. The other study represents the only published clinical trial to investigate the therapeutic potential of CBD to treat cognitive impairment in a neuropsychiatric condition. Unfortunately, it failed to show a significant cognitive improvement in schizophrenia patients treated with CBD [63].

### Cognitive Function in Cannabis Users Using Products with a High or Low Δ9-THC:CBD Ratio

The relative ratio of Δ9-THC and CBD varies greatly in cannabis available on the street. For instance, in the UK, more recent varieties of cannabis, such as “skunk,” have been shown to contain more Δ9-THC and virtually no CBD compared to the traditional “hash” variety [64]. A shift towards use of high-potency cannabis with higher levels of Δ9-THC and lower levels of CBD has been reported also in the USA [65] and Australia [66]. In 2012, a meta-analysis of the studies published over the previous three decades indicated that there has been a consistent increase in cannabis potency worldwide [67]. Recent research has indicated that the use of skunk-like high-potency cannabis might have a more detrimental effect on mental health than the use of the traditional low-potency cannabis [5, 42], suggesting that the harmful effect of cannabis might be driven by the high Δ9-THC/low CBD contained in the used strain. However, despite being consistent so far, evidence is too limited to draw definitive conclusions.

In a naturalistic study [51], Morgan and colleagues investigated cognitive processing among cannabis users as a function of the CBD content in the cannabis strain used by the study participants. Results indicate that, upon acute intoxication, participants using low-CBD cannabis (<0.14%; *n* = 22) performed worse than those using high-CBD cannabis (>0.75%; *n* = 22) on a verbal memory task, despite there being no difference in the concentration of Δ9-THC present in the cannabis used during the experiment or in the baseline (before acute intoxication) verbal memory performance between the low- and high-CBD cannabis-using groups. Moreover, this effect was still significant when controlling for the confounding effect of alcohol use and intelligence. Together, these results suggest that CBD may attenuate the acute memory-impairing effects of Δ9-THC.

In a second experiment, Morgan and colleagues assessed cognitive functioning in chronic cannabis users when they were not intoxicated as a function of the Δ9-THC:CBD ratio in the strain of cannabis they used [50]. Results revealed a better recognition memory in subjects using high-CBD cannabis compared to that in subjects using low-CBD cannabis. Also, this association was independent of the frequency of use (recreational use, *n* = 54; daily use, *n* = 66). Further analysis also suggested an association between daily use of high Δ9-THC cannabis and disruption of verbal and episodic memory. Altogether, evidence from these studies suggests a higher risk of memory disruption associated with consuming cannabis strains with high Δ9-THC and low CBD content.

### Cognitive Function in Healthy Subjects Following Administration of Pure Δ9-THC and CBD

A number of studies have investigated the effects of the major cannabinoids such as Δ9-THC and CBD administered as pure pharmacological grade substance to avoid the confounding effects of different CBD and Δ9-THC concentrations and other cannabinoids present in the cannabis extract. This research design, which has been successfully used in preclinical models, represents a promising paradigm in understanding the neurobiological mechanisms underlying the effects of cannabinoids on cognition [40].

Hindocha and colleagues investigated the main effects and interaction of Δ9-THC and CBD on facial emotion recognition [53••]. Cannabis-using participants (*n* = 48) received Δ9-THC, CBD, or their combination (Δ9-THC + CBD) over separate sessions. Results indicate a detrimental effect of administration of Δ9-THC alone on emotional processing accuracy, which was no longer present when participants received the Δ9-THC + CBD combination. Furthermore, administration of CBD alone was associated with higher emotional processing accuracy compared to placebo. This study also revealed that the effects of Δ9-THC and CBD on emotional processing were not affected by the participants’ frequency of cannabis use or schizotypal traits.

Using a slightly different approach, another study investigated the putative protective effect of CBD against Δ9-THC-induced impairments in cognitive processing [52••]. Healthy participants were randomized to receive CBD or placebo before being intravenously administered with Δ9-THC. Compared to the CBD pretreatment group (*n* = 22), the placebo pretreatment group (*n* = 26) showed a poorer verbal memory performance following Δ9-THC administration. Moreover, while Δ9-THC affected working memory performance in participants pretreated with placebo, pretreatment with CBD was able to partially prevent the detrimental effects of Δ9-THC on this cognitive domain.

### Cognitive Function in Healthy Subjects Following Administration of Standardized Cannabis Extracts with Defined Δ9-THC:CBD Ratios

A few studies have investigated the effect of standardized cannabis extracts containing precise combinations of Δ9-THC and CBD*.* This approach complements studies investigating the effects of pure pharmacological grade cannabinoids by shedding new light on the potential cognitive mechanisms that may underpin the harmful effects of specific types of recreational cannabis [5, 42].

Roser and colleagues investigated whether the acute effect of standardized cannabis extracts on selective attention varies as a function of Δ9-THC being administered alone or in combination with CBD [54]. Results indicate that cognitive performance among healthy participants (*n* = 20) did not differ significantly when administered with Δ9-THC alone or in combination with CBD. However, compared to placebo, both Δ9-THC alone and combined Δ9-THC and CBD administration affected the attention-related electrophysiological response. Another study assessed attention and working memory, exposing recreational cannabis users to increasing dosages of Δ9-THC alone or to cannabis extracts containing increasing balanced dosages of both Δ9-THC and CBD [55]. Results suggest that higher doses of Δ9-THC were associated with longer latency for short-term memory. Interestingly, this effect on working memory was no longer present when participants received any of the cannabis extracts containing both Δ9-THC and CBD.

### Cognitive Function in Individuals Treated with Medical Marijuana

So far, only two studies have investigated the effect of MMJ on cognition. Wade and colleagues tested for the effect of Δ9-THC, CBD, and a MMJ product containing a 1:1 Δ9-THC:CBD ratio in patients with different neurological disorders, mainly multiple sclerosis (*n* = 24) [57]. Administration of Δ9-THC alone affected cognition in patients, as assessed with a Short Orientation Memory Concentration test. Despite no main effect of CBD on its own, when administered in combination with Δ9-THC in the MMJ product, it prevented the Δ9-THC-induced detrimental effect on cognition. More recently, a pilot study has assessed the potential effect of a 3-month treatment with MMJ on executive function [56]. Despite the modest sample size (*n* = 11), patients certified for MMJ use experienced some improvement on measures of executive function, mostly reflecting an increased processing speed with preserved task accuracy.

### Neural Substrates of Human Cognition Modulated by Administration of Pure Δ9-THC and CBD

Over the years, imaging studies investigating how cannabinoids modulate human brain function have employed progressively more sophisticated designs. Recent studies have allowed investigation of the effect of acute cannabinoid administration on neural networks underlying specific cognitive domains and their relationship to any concomitant effect on cognitive task performance and neuropsychiatric symptomatology induced by the cannabinoids. This body of research has complemented the behavioral evidence, testing specific hypotheses for a role of the endocannabinoid system in various cognitive processes.

Bhattacharyya and colleagues directly contrasted for the first time the effects of Δ9-THC and CBD administration on brain function and related behavior (*n* = 15) in healthy individuals, reporting opposite effects relative to placebo across different cognitive domains [58••]. More specifically, during the retrieval condition of a verbal memory task, CBD acutely increased activation of the striatum and prefrontal cortex relative to the placebo condition. Conversely, Δ9-THC reduced activity in these regions and the reduced striatal activation correlated with the severity of the psychotic symptoms induced by it. Both Δ9-THC and CBD modulated the activation of the amygdala and related brain regions involved in fear processing, which correlated with their opposing effects on anxiety, with effects of Δ9-THC correlating with increase in anxiety symptoms and fear-related autonomic arousal induced by it and effects of CBD correlating with a reduction in anxiety and attenuation of fear-related autonomic arousal under its influence. During a response inhibition task, activation in the parahippocampal gyrus bilaterally and in the left insula and caudate was attenuated following Δ9-THC administration, but enhanced under CBD. Finally, Δ9-THC and CBD induced opposite effects while processing a response inhibition task and auditory and visual stimuli.

In another study from the same sample, the authors reported opposite effects of Δ9-THC and CBD administration relative to that of placebo on several brain areas during attentional salience processing [60]. More specifically, Δ9-THC acutely reduced striatal activation which correlated with the severity of the Δ9-THC-induced psychotic symptoms as well as the Δ9-THC-induced behavioral response latency. A recent investigation in the same sample compared the effects of Δ9-THC and CBD administration on functional connectivity during the salience processing task [61••]. In line with previous reports, the two cannabinoids had opposite effects on functional connectivity between the dorsal striatum, the prefrontal cortex, and the hippocampus. Specifically, mediotemporal-prefrontal connectivity was enhanced under the Δ9-THC condition but reduced following CBD administration. Instead, fronto-striatal connectivity was enhanced by CBD but reduced under Δ9-THC. The effect of Δ9-THC on fronto-striatal connectivity also correlated with response latency while performing the task.

Another study has directly compared the effects of Δ9-THC and CBD administration on the processing of auditory and visual stimuli [59]. Compared to CBD, while processing auditory stimuli, Δ9-THC administration was associated with reduced activity in the right superior and middle temporal gyri. During the visual processing condition, Δ9-THC increased activity in the primary visual cortex (left lingual and middle occipital gyri) but attenuated it in occipital regions bilaterally compared to CBD. Δ9-THC and CBD also had opposite effects on cerebellar activity during visual processing.

### Cognitive Function in Healthy Subjects Following Administration of Pure Δ9-THC and Δ9-THCV

To date, human studies have typically focused on Δ9-THC and CBD, and their effects on brain function have been extensively studied and well understood [41]. Only one recent study has investigated the effect of another cannabinoid, Δ9-THCV, a cannabinoid receptor 1 (CB1) neutral antagonist, on cognition in humans [62]. Englund and colleagues assessed the acute effects of intravenous Δ9-THC administration on cognition in healthy subjects (*n* = 10) following pretreatment with Δ9-THCV or placebo for 5 days. Upon acute Δ9-THC administration, participants pretreated with placebo experienced an impairment in delayed verbal memory recall, which was no longer present when participants were investigated following pretreatment with Δ9-THCV. However, compared to the placebo pretreatment condition, Δ9-THC administration following Δ9-THCV pretreatment was associated with significantly increased memory intrusions.

## Discussion

### A Promising Approach to Understanding the Effects of Cannabinoids on Cognition

Several potential confounders limit any inference being drawn from studies investigating the association between chronic or occasional cannabis exposure and cognitive processing alterations. These include (i) considerable variation in the ratio of different cannabinoids, in particular Δ9-THC:CBD ratio, in cannabis used recreationally; (ii) interindividual variation in frequency, quantity, and duration of cannabis use; (iii) modality of cannabis use, frequently consumed in combination with tobacco and/or other substances; and (iv) neuroadaptive changes that occur in relation to tolerance, withdrawal and/or sensitization, and substance abuse/dependence in general. In an attempt to control for these factors, several studies have investigated the effect of each of these cannabinoids separately and contrasted them directly to determine whether the composition of cannabis matters in terms of its effects on human cognition. These pharmacological challenge studies, which we have reviewed here, offer the unique possibility of perturbing the endocannabinoid system under controlled experimental conditions and investigating their effect on various cognitive processes as well as acutely model cannabis-related neuropsychiatric manifestations. This line of research also allows us to systematically investigate under controlled experimental conditions the therapeutic potential or indeed the harmful effects of different cannabinoids that are increasingly being considered as treatment for various neurodegenerative and neuropsychiatric disorders. This research approach, especially when combined with neuroimaging techniques, can provide insight into the neurobiological mechanisms underlying the effects of cannabis on human cognition [11].

### Cannabis Composition and Altered Cognitive Processing: What and Where is the Evidence

Most of the available evidence so far relates to pharmacological challenge studies involving two cannabinoids, ∆9-THC and CBD, the most known and investigated cannabinoids at a neurobiological level and in preclinical models [40]. Using different research strategies, these two cannabinoids have been administered as a crude extract (pure form), or in different concentrations within recreational cannabis, standardized cannabis extracts, or medicinal cannabis preparations.

#### Effect of Δ9-THC on Human Cognition and Related Neural Activity

Compared to studies assessing the long-lasting effects of cannabis use on cognition [16, 18, 46, 68–70], there is much better agreement across systematic experimental studies that ∆9-THC administration acutely impairs several cognitive domains, including emotional processing [53••], and verbal [52••, 62] and working memory performance [52••]. Moreover, imaging studies have extended this evidence, indicating acute ∆9-THC-induced changes in the neural activity of brain areas underlying verbal memory, emotional and attentive processing, and processing of visual and auditory stimuli [58••, 59, 60, 61••]. Intriguingly, under the acute effect of ∆9-THC, changes in these neural networks were associated with the acute manifestation of psychotic symptoms [58••, 59, 60], anxiety [58••], and impaired cognition at a behavioral level [61••].

#### Effect of CBD on Human Cognition and Related Neural Activity

Over the last few years, studies have also investigated the effect of acute CBD administration on cognition, in quest of a potential therapeutic effect of this cannabinoid [58••]. This has followed evidence that CBD may be an antagonist/inverse agonist at cannabinoid receptor type 1 (CB1), in opposition to Δ9-THC partial agonist effect [71], and additional evidence for a potential anxiolytic effect of CBD, based on preclinical [72–76] and clinical [77, 78] studies. Limited evidence so far suggests that CBD administration might improve emotional processing accuracy in cannabis users [53••]. Evidence from imaging studies suggests that CBD attenuates amygdala activity while processing fearful stimuli, which was associated with an attenuation of anxiety and the normal autonomic arousal associated with fear processing [58••], consistent with independent evidence of its anxiolytic potential [79]. On the contrary, CBD administration did not improve cognitive functioning in individuals suffering from neurological disorders [57]. However, most of this evidence is based on single studies, which need independent replication.

#### Comparison of the Effect of Δ9-THC and CBD on Human Cognition and Related Neural Activity

Studies have investigated the effects of both ∆9-THC and CBD, in their pure forms or in different combinations, in order to test whether CBD may protect from or reverse the adverse effects of ∆9-THC on cognition. Evidence from the studies reviewed here seems to suggest the following: (i) Acute cannabis-induced detrimental effects on verbal memory may be greater when exposed to low-CBD cannabis [51]; (ii) Chronic cannabis use may be associated with poorer verbal and episodic memory when exposed to low CBD/high ∆9-THC cannabis [50]; (iii) Acute Δ9-THC-induced detrimental effects on emotional processing and working memory are absent if Δ9-THC is administered in combination with CBD [53••, 55]; (iv) Acute effects of Δ9-THC on attention may not be distinguishable from acute effects of Δ9-THC administered in combination with CBD [54]; (v) Pretreatment with CBD may prevent the acute Δ9-THC-induced impairments in verbal and working memory processing [52••]; (vi) Acute Δ9-THC-induced impairments in cognition in patients with neurological disorders may be absent if Δ9-THC is administered in combination with CBD in medicinal cannabis preparation [57]; (vii) Medicinal cannabis, containing both Δ9-THC and CBD, may have a beneficial effect on certain cognitive processes in patients certified for its use [56]; (viii) Δ9-THC and CBD have opposite effects on neural activity in brain areas related to several cognitive processes [58••, 59, 60, 61••]; (ix) Pretreatment with Δ9-THCV may prevent some of the acute Δ9-THC-induced detrimental effects on verbal memory. However, it is worth highlighting the need for independent replication of evidence summarized here before they may be generalized and useful for application in clinical settings.

### Limitations and Future Perspectives

Studies included in this review need to be considered in light of some limitations. On the basis of current evidence, it is perhaps premature to conclude that any protective cognitive effects in naturalistic studies are wholly attributable to CBD, considering the numerous other chemicals present in cannabis [50, 51]. It is unclear from existing research whether there is a specific ratio of Δ9-THC and CBD at which the beneficial effects of CBD outweigh any harmful effects of Δ9-THC on cognition, such that this ratio may serve as a threshold for consideration in the formulation of medicinal cannabis preparations [56]. Whether Δ9-THCV has any beneficial effect is unclear at this stage due to its mixed effects on memory and also because current evidence comes from a single human study with a modest sample size [62]. Research in humans, especially on the effect of CBD and other cannabinoids, is still in its infancy. So far, the only published study on the acute effect of CBD in schizophrenia has not shown any significant cognitive improvement [63]. Promising results of beneficial effects of CBD on cognition in the context of neuropsychiatric diseases come from preclinical studies on Alzheimer’s disease (AD), indicating improved recognition, social recognition, and spatial memory in AD paradigms following acute and chronic CBD treatment (reviewed in [80]). To our knowledge, no studies have explored the effect of CBD on cognition in other drug-induced states. Future investigations need to investigate the effect of different cannabinoids on human cognition to better understand their therapeutic potential as well as relevance for neuropsychiatric conditions where the endocannabinoid system or cannabis exposure may play a role [12, 41, 42, 81–84].

## Conclusions

Available evidence suggests that acute Δ9-THC administration has an adverse effect on several cognitive domains. In particular, memory components appear to be the cognitive domains more consistently disrupted following acute Δ9-THC administration, including verbal, episodic, and working memory. Less strong evidence suggests a deleterious effect of Δ9-THC on attention and emotional processing. Furthermore, the detrimental effects of cannabis exposure on cognition appear to be driven by Δ9-THC, with preparations containing a high Δ9-THC:CBD ratio causing greater impairments in emotional processing and memory function compared to preparations with low Δ9-THC:CBD ratio. Limited evidence also suggests potential beneficial effects of CBD alone on emotional processing and some protective effects of CBD against Δ9-THC-induced impairments in emotional processing and memory function. Moreover, Δ9-THC and CBD appear to have antagonistic effects on neural networks underlying several cognitive processes, some of which correlate with the harmful (e.g., Δ9-THC-induced psychotic or anxiety symptoms) or beneficial (e.g., anxiolytic effect of CBD) effects of these cannabinoids on behavior.
